# Community Stakeholders’ Perceptions of Barriers to Childhood Obesity Prevention in Low-Income Families, Massachusetts 2012–2013

**DOI:** 10.5888/pcd12.140371

**Published:** 2015-03-26

**Authors:** Claudia Ganter, Emmeline Chuang, Alyssa Aftosmes-Tobio, Rachel E. Blaine, Mary Giannetti, Thomas Land, Kirsten K. Davison

**Affiliations:** Author Affiliations: Emmeline Chuang, University of California, Los Angeles, Los Angeles, California (Dr. Chuang was at University of California, San Diego, at the time this article was written); Alyssa Aftosmes-Tobio, Rachel E. Blaine, Kirsten K. Davison, Harvard School of Public Health, Boston, Massachusetts; Mary Giannetti, Montachusett Opportunity Council, Fitchburg, Massachusetts; Thomas Land, Massachusetts Department of Public Health, Boston, Massachusetts; Ms. Ganter is also affiliated with the Technical University of Berlin, Department of Health Care Management, Berlin, Germany.

## Abstract

**Introduction:**

The etiology of childhood obesity is multidimensional and includes individual, familial, organizational, and societal factors. Policymakers and researchers are promoting social–ecological approaches to obesity prevention that encompass multiple community sectors. Programs that successfully engage low-income families in making healthy choices are greatly needed, yet little is known about the extent to which stakeholders understand the complexity of barriers encountered by families. The objective of this study was to contextually frame barriers faced by low-income families reported by community stakeholders by using the Family Ecological Model (FEM).

**Methods:**

From 2012 through 2013, we conducted semistructured interviews with 39 stakeholders from 2 communities in Massachusetts that were participating in a multisector intervention for childhood obesity prevention. Stakeholders represented schools; afterschool programs; health care; the Special Supplemental Nutrition Program for Women, Infants, and Children; and early care and education. Interviews were audio-recorded, transcribed, coded, and summarized.

**Results:**

Stakeholder reports of the barriers experienced by low-income families had a strong degree of overlap with FEM and reflected awareness of the broader contextual factors (eg, availability of community resources, family culture, education) and social and emotional dynamics within families (eg, parent knowledge, social norms, distrust of health care providers, chronic life stressors) that could affect family adoption of healthy lifestyle behaviors. Furthermore, results illustrated a level of consistency in stakeholder awareness across multiple community sectors.

**Conclusion:**

The congruity of stakeholder perspectives with those of low-income parents as summarized in FEM and across community sectors illustrates potential for synergizing the efforts necessary for multisector, multilevel community interventions for the prevention of childhood obesity.

## MEDSCAPE CME

Medscape, LLC is pleased to provide online continuing medical education (CME) for this journal article, allowing clinicians the opportunity to earn CME credit.

This activity has been planned and implemented in accordance with the Essential Areas and policies of the Accreditation Council for Continuing Medical Education through the joint sponsorship of Medscape, LLC and Preventing Chronic Disease. Medscape, LLC is accredited by the ACCME to provide continuing medical education for physicians.

Medscape, LLC designates this Journal-based CME activity for a maximum of 1 ***AMA PRA Category 1 Credit(s)™***. Physicians should claim only the credit commensurate with the extent of their participation in the activity.

All other clinicians completing this activity will be issued a certificate of participation. To participate in this journal CME activity: (1) review the learning objectives and author disclosures; (2) study the education content; (3) take the post-test with a 75% minimum passing score and complete the evaluation at www.medscape.org/journal/pcd; (4) view/print certificate.

**Release date: March 26, 2015; Expiration date: March 26, 2016**

### Learning Objectives

Upon completion of this activity, participants will be able to:

- Assess perceived barriers to treating child obesity in the domain of family history and structure
- Analyze stakeholder perceptions regarding cultural barriers to reducing childhood obesity
- Assess perceived community barriers to treating child obesity
- Evaluate other variables that might impede the care of children with obesity

**EDITORS**

Rosemarie Perrin, Editor, *Preventing Chronic Disease*. Disclosure: Rosemarie Perrin has disclosed no relevant financial relationships.

**CME AUTHOR**

Charles P. Vega, MD, Clinical Professor of Family Medicine, University of California, Irvine. Disclosure: Charles P. Vega, MD, has disclosed the following relevant financial relationships: Served as an advisor or consultant for: Lundbeck, Inc.; McNeil Pharmaceuticals; Takeda Pharmaceuticals North America, Inc.

**AUTHORS AND CREDENTIALS**

Disclosures: Claudia Ganter, MPH; Emmeline Chuang, PhD; Alyssa Aftosmes-Tobio, MPH; Rachel E. Blaine, MPH, RD; Mary Giannetti, MS, RD; Thomas Land, PhD; Kirsten K. Davison, PhD have disclosed no relevant financial relationships.

Affiliations: Claudia Ganter, MPH, Department of Nutrition, Harvard School of Public Health, Boston, MA; Emmeline Chuang, University of California, Los Angeles, Los Angeles, California; Alyssa Aftosmes-Tobio, Rachel E. Blaine, Kirsten K. Davison, Harvard School of Public Health, Boston, Massachusetts; Mary Giannetti, Montachusett Opportunity Council, Fitchburg, Massachusetts; Thomas Land, Massachusetts Department of Public Health, Boston, Massachusetts; Ms. Ganter is also affiliated with the Technical University of Berlin, Department of Health Care Management, Berlin, Germany.

## Introduction

Approximately 1 in 5 children and adolescents in the United States aged 2 to 19 years is obese (1). Although 2011–2012 data from the National Health and Nutrition Examination Survey (NHANES) illustrates that obesity among preschoolers has significantly decreased from previous estimates, racial/ethnic and socioeconomic disparities in childhood obesity persist (1,2). The etiology of childhood obesity is multidimensional and includes familial, organizational, and societal factors. To more effectively address these factors, policymakers and researchers are increasingly promoting social ecological approaches to obesity prevention that encompass multiple community sectors (3–8). Multi-sector approaches require stakeholders to work collaboratively across community sectors to develop and sustain programs that influence policies, systems, and environments in ways that make it easier for families to make healthy lifestyle choices, such as purchasing and consuming healthful food and engaging in physical activity such as outdoor play.

To effectively support behavior change through a synergistic, multisector approach, stakeholders in participating sectors must be aware of the complex and multifaceted barriers to behavior change encountered by families, particularly low-income families (9–13). Gaps in stakeholder awareness within or across sectors would compromise the concentrated effort needed to prevent obesity; these gaps indicate the need for additional training. However, to our knowledge, the extent to which stakeholders are informed about families’ experiences in relation to obesity prevention has not been documented in obesity prevention research.

To address this gap, we interviewed key stakeholders across 5 community sectors: primary health care providers (health care); the Special Supplemental Nutrition Program for Women, Infants, and Children (WIC); early care and education (early education), schools, and afterschool programs. Our objectives were to 1) characterize stakeholders’ perceptions of the barriers low-income parents experience in the context of obesity prevention, 2) examine the extent to which stakeholders’ perceptions align with parents’ perceptions as documented in the Family Ecological Model (FEM); and 3) assess variations in stakeholders’ perceptions by community sector.

The FEM was used to guide this study (Figure). FEM was developed to support and guide research in childhood obesity prevention and has been validated with a cohort of low-income families with preschool-aged children via an in-depth qualitative approach (14,15). Consistent with ecological systems theory (16), FEM outlines contextual and family systems factors that influence children’s diets, physical activity, and screen-based behaviors and highlights the importance of engaging families in obesity prevention strategies across community sectors. Although FEM includes 4 temporally organized dimensions, this study focused on the 2 dimensions most relevant to understanding the broader life factors that may inhibit healthy lifestyle behaviors in low-income families, Family Ecology and Family Social and Emotional Context. Family Ecology encompasses contextual factors that influence behavior, such as family history and structure, organizational characteristics, community characteristics, and media and policy factors. Family Social and Emotional Context results from the family ecology and includes family knowledge and social norms as well as social disparities and chronic stress. The other 2 FEM dimensions were omitted because they focus on outcomes rather than determinants of behavior change.

**Figure:**
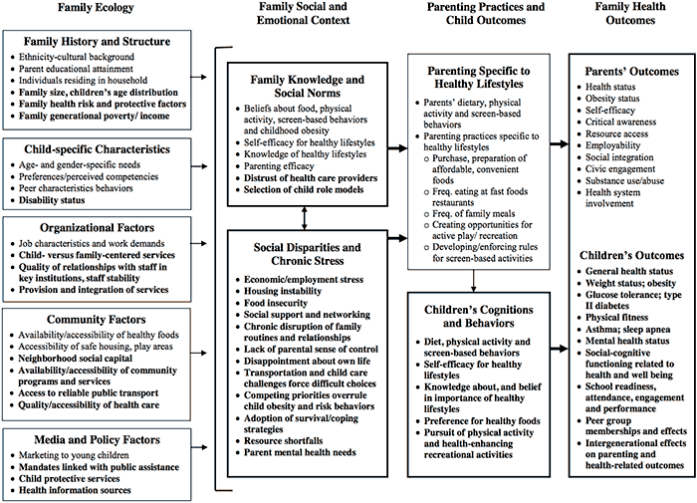
The Family Ecological Model. Reprinted with permission from Davison KK, Jurkowski JM, Lawson HA. Reframing family-centred obesity prevention using the Family Ecological Model. Public Health Nutr 2013;16(10):1861-9.

**Table:** 

The Family Ecological Model (FEM) is divided into 4 vertical sections labeled: Family Ecology, Family Social and Emotional Context, Parenting Practices and Child Outcomes, and Family Health Outcomes. The sections flow stepwise from left to the right, starting with the first 2 sections, which list the most important, broader life factors that may inhibit healthy lifestyle behaviors in low-income families. The first section is divided into 5 boxes:
1. Family History and Structure:
Ethnicity-cultural background;
Parent educational attainment; Individuals residing in household;
Family size, children’s age distribution;
Family health risk and protective factors; and
Family generational poverty/income.
2. Child-specific Characteristics:
Age- and gender-specific needs;
Preferences/perceived competencies;
Peer characteristic behaviors; and
Disability status,
3. Organizational Factors:
Job characteristics and work demands;
Child- versus family-centered services;
Quality of relationships with staff in key institutions, staff stability; and
Provision and integration of services.
4. Community Factors:
Availability/accessibility of healthy foods;
Accessibility of safe housing, play areas;
Neighborhood social capital;
Availability/ accessibility of community programs and services;
Access to reliable public transport, and
Quality/accessibility of health care.
5. Media and Policy Factors:
Marketing to young children;
Mandates linked with public assistance;
Child protective services; and
Health information sources.
From each of the 5 boxes a 1-way horizontal arrow runs to the second section, Family Social and Emotional Context, which results from the Family Ecology section. This section has 2 boxes that are connected with a 2-way vertical arrow:
1. Family Knowledge and Social Norms, with the following subdomains:
Beliefs about food, physical activity, screen-based behaviors and childhood obesity;
Self-efficacy for healthy lifestyles;
Knowledge of healthy lifestyles;
Parenting efficacy;
Distrust of health care providers; and
Selection of child role models.
2. Social Disparities and Chronic Stress, with the following subdomains:
Economic/employment stress;
Housing instability;
Food insecurity;
Social support and networking;
Chronic disruption of family routines and relationships;
Lack of parental sense of control;
Disappointment about own life;
Transportation and child care challenges force difficult choices;
Competing priorities overrule child obesity and risk behaviors;
Adoption of survival/coping strategies;
Resource shortfalls; and
Parent mental health needs.
Each box is connected with a 1-way horizontal arrow to the first box in the third section,
3. Parenting Practices and Child Outcomes. The third section consists of 2 boxes:
Parenting Specific to Healthy Lifestyles and includes the following subdomains:
Parents’ dietary, physical activity and screen-based behaviors and
Parenting practices specific to healthy lifestyles, with:
Purchase, preparation of affordable, convenient foods;
Frequency of eating at fast food restaurants;
Frequency of family meals;
Creating opportunities for active play/recreation; and
Developing/enforcing rules for screen-based activities. This box connects by a one-way vertical arrow to the box underneath called Children’s Cognitions and Behaviors, which has the following subdomains:
Diet, physical activity and screen-based behaviors;
Self-efficacy for healthy lifestyles;
Knowledge about, and belief in importance of healthy lifestyles;
Preference for healthy foods; and
Pursuit of physical activity and health-enhancing recreational activities.
4. Family Health Outcomes, the last section, results from all the other 3 sections. Two boxes from the third section are each connected to the last section by a 1-way horizontal arrow and have the following subdomains:
Parents’ Outcomes:
Health status;
Obesity status;
Self-efficacy; Critical awareness;
Resource access; Employability;
Social integration; Civic engagement;
Substance use/abuse; and
Health system involvement.
Children’s Outcomes are:
General health status;
Weight status, obesity;
Glucose tolerance, type II diabetes;
Physical fitness;
Asthma, sleep apnea;
Mental health status;
Social-cognitive functioning related to health and well-being;
School readiness, attendance, engagement and performance;
Peer group memberships and effects; and
Intergenerational effects on parenting and health-related outcomes.

## Methods

### Setting

This study is nested in the Massachusetts Childhood Obesity Research Demonstration study (MA-CORD), a multisector community intervention for childhood obesity prevention that is being implemented in 2 low-income Massachusetts communities (17,18). Both communities are small- to mid-size (population 40,000–100,000) with predominantly non-Hispanic white (~68%) and sizeable Hispanic (16%–21%) populations. Mean income per capita is approximately $22,000 (state average is $35,000) and rates of poverty range from 23% to 27% (state poverty rate is 12%). MA-CORD includes childhood obesity interventions in 5 sectors, including afterschool programs, schools, health care, WIC, and early education.

### Participants

Eligible stakeholders were drawn from the 5 community sectors in both MA-CORD communities. All stakeholders who participated in an introductory meeting about MA-CORD were invited to participate in a semistructured interview. Stakeholders who agreed to participate were sent an email after the meeting. Any stakeholders who did not reply after 3 reminders were counted as nonresponders. Of 108 stakeholders who were eligible to participate, 71 were from schools, 17 from afterschool programs, 9 from health care, 6 from WIC, and 5 from early education. A total of 63 stakeholders (58.3% of those eligible) agreed to participate in the study, and 39 stakeholders (61.9% of 63) were interviewed.

### Interview procedures

All interviews were conducted at baseline, after the organizations agreed to participate in MA-CORD but before the implementation of childhood obesity intervention activities. Some training sessions for stakeholders had already been delivered by the time of the interview. Eight stakeholder interviews were conducted face-to-face and 31 by telephone. Two of the authors (C.G. and A.A.) conducted the interviews from September 2012 through March 2013. To mediate potential differences in interview style, an interview guide was developed, and the first 3 interviews were conducted with both interviewers present. All participants gave permission for audio recording. All procedures were reviewed and approved by the institutional review boards of Harvard School of Public Health and San Diego State University. Stakeholders were compensated with a $10 gift card.

### Data analysis

Interviews were transcribed verbatim and entered into NVivo version 10.0 (QSR International). Data were analyzed through coding and extraction (19). An initial list of codes and definitions was developed on the basis of the Family Ecology and Family Social and Emotional Context domains of FEM (columns 1 and 2 in the Figure). Two investigators (C.G. and R.E.B.) pilot-tested this initial list with 5 randomly chosen interviews, which was refined and expanded to incorporate emergent themes. The investigators then independently coded 5 more interviews with the revised scheme. Disagreements in coding were discussed. A consensus meeting with a third investigator (K.K.D.) was held to finalize the coding scheme. Data analysis focused on the extent to which stakeholder descriptions of barriers experienced by low-income families reflected the experiences of low-income families as documented in the FEM model. Coded data were analyzed for potential differences in stakeholder perceptions and sector.

## Results

The 39 stakeholders interviewed represented all sectors of MA-CORD. Fifteen stakeholders were from schools, 8 from afterschool programs, 7 from health care, 6 from WIC, and 3 from early education. Participant characteristics are summarized in Table 1.

**Table 1:** Characteristics of Participating Community Stakeholders (N = 39), Study of Community Stakeholders’ Perceptions of Barriers to Childhood Obesity Prevention for Low-Income Families, Massachusetts, 2012–2013

Stakeholder Characteristic	N (%)
**Community sector**	
Primary health care	7 (18.0)
Special Supplemental Nutrition Program for Women, Infants and Children	6 (15.4)
Schools	15 (38.5)
Afterschool programs	8 (20.5)
Early care and education	3 (7.7)
**Organizational role**	
Implementer	30 (76.9)
Program leader	9 (23.1)
**Participation rate**	
Invited to participate	108 (100.0)
Agreed to be interviewed	63 (58.3)
Completed interviews	39 (61.9)
**Sex**	
Female	36 (92.3)
Male	3 (7.7)
**Age, y**	
18–29	5 (12.8)
30–39	9 (23.0)
40–49	11 (28.2)
50–59	12 (30.8)
≥60	2 (5.1)
**Ethnicity**	
Hispanic	5 (12.8)
Not Hispanic	34 (87.2)
**Race**	
White	33 (84.6)
Asian	2 (5.1)
African American	1 (2.6)
Unknown	3 (7.7)
**Interview length, min**	
Average	24
Shortest	10
Longest	64

Stakeholders described a wide range of barriers affecting healthy lifestyle choices among low-income families. Almost all barriers identified by stakeholders could be categorized within the Family Ecology and Family Social and Emotional Context domains of FEM. We summarized the major themes identified by stakeholders in each of the 2 domains addressed in the model and described differences in stakeholder perspectives by sector, including illustrative quotations (Table 2).

**Table 2:** Study of Community Stakeholders’ (N = 39) Perceptions of Barriers to Childhood Obesity Prevention for Low-Income Families, Massachusetts, 2012–2013: Quotes Illustrating Opinions, by Theoretical Domain

Family Ecological Model Construct Category, n (%)^a^	Community Stakeholder Sector	Quote
**Family Ecology**		
Family history and structure: ethnicity–cultural background, 32 (82%)	Primary health care	1. “Particularly immigrant families . . . they’re impoverished here but they were even more impoverished back home, um, that you know there’s a certain amount of pride in being able to feed your child and have a chubby little kid. And I think that’s a real challenge, to get past that you know, a chubby kid is a healthy kid concept that I think many of our immigrant families have.”
	WIC	2. “You know, fat babies are healthy babies in the Spanish culture.”
	WIC	3. “The grandparents want the grandchild to be plump. You know. Um, so it’s a lot of I think a lot has to do culturally. They want that child to be large and big and plump.”
	Primary health care	4. “The grandparents are a big issue in terms of undermining some of the efforts to get a child to a healthy weight.”
	Afterschool programs	5. “As we were talking about it [food intake], his grandmother was sitting next to us, and she interrupted us and she said: ‘He’s a bambino. He needs to be able to eat whatever he wants.’”
Family history and structure: parent educational attainment, 13 (33%)	Primary health care	6. “We also have families that have parents that don’t read or write. Most of the information sometimes we give out, it’s all written material.”
	Primary health care	7. “I always knew that the literacy level was low within our population but that just underscored it by the number of patients who even though we have the forms in both English and Spanish cannot complete the form. So there’s a fairly significant number of people who do not read, or read at a very low grade level in terms of the parents . . . If you have patients who can’t read they are not going to be able to look at the nutrition information on a food item.”
	School	8. “Sometimes people . . . [are] English language learners because they can’t speak, you know I would say a lot of Spanish families don’t really get involved. Not that they don’t care about their children but because of the communication.”
	Afterschool programs	9. “I offered a nutritional class, and . . . everything I did was in English and Spanish, and I had a Spanish translator come in. All my handouts were in Spanish and English . . . and no one showed up. None of the parents came in, none of them. There are so many obstacles in the city.”
Organizational factors: quality of relationship with provider,^b^ 15 (39%)	School	10. “Basically the challenge is not to get them defensive. And to have them feel that I’m on their side . . . I’m not trying to be critical. That is a big challenge. Because it’s a very sensitive issue.”
	Afterschool programs	11. “It is a reality check and not many people can handle that.”
	WIC	12. “So if you talk more about health than about being overweight then I think they are more receptive, because you’re not targeting them personally.”
Community factors: accessibility of safe housing, play areas, 19 (49%)	Childcare	13. “I think its activity, I think a lot of it is parents are afraid to let their children go out and play.”
	Primary health care	14. “First is availability of safe physical activity. There are certain areas of [community name] where parents rightly so don’t want their kids going outside to play. There may not be safe spaces for them to play and in terms of the way traffic and things. But beyond that there is significant gang gun violence in our city as well.”
Community Factors: Access to reliable public transport, 16 (41%)	Primary health care	15. “Almost nobody I take care of has a car. When you say, ‘Join the soccer league,’ they are like, ‘In your dreams.’”
	School	16. “Transportation, you know to get them to take them. You can offer a free program, that’s great, but can the families get there.”
Community factors: availability or accessibility of healthful and unhealthful foods, 15 (39%)	Early care and education	17. “We actually only have three grocery stores . . . for a large city that’s a very small number. . . . It’s those convenience stores that are on the corners and are near the housing. . . . They are offering candy and chips and everything else, and where are the fruits and the vegetables and everything else that we preach about? But they [parents] don’t have the accessibility to find and even purchase some of those items.”
	School	18. “They [parents] are trying to feed what’s affordable, and sometimes what’s affordable isn’t always the best choice.”
Media and policy factors: marketing, 7 (18%)	Afterschool programs	19. “I think the first one that always comes to mind, parents find or there is almost a false perception that healthy eating is expensive. You know there are ways to work around that, to get foods that are healthy and inexpensive but I think as a society we’ve made it out to be that healthy foods are, they’re expensive. You have to go to Whole Foods and you have to spend $10 on apples like I mean, it’s there is that sort of perception. So I think that’s the biggest barrier for families is they think that they can’t, they don’t have the funds for it.”
	Early care and education	20. “I think the parents, I think a lot of them don’t know, they um, you know, they see advertisements and everything looks healthy and natural and they’re not.”
**Family Social and Emotional Context**		
Family knowledge and social norms: knowledge of healthy lifestyles, 13 (33%)	School	21. “I don’t think the parents understand what obesity is because they’re obese, so they need to be educated on how to make healthy meals and stuff.”
	School	22. “If parents understand how they can make substitutions in the diet in a way that’s economical, so that they can afford to put better nutritious meals on the table, parents would do that. Particularly when they understand the connection between what the children eat and their overall health and well-being.”
	Primary health care	23. “They know McDonald’s is not good, but they don’t know what they are buying at home also seem as McDonald’s. They know fast food is not good, but they don’t know exactly what food is fast food, what food is . . . the healthier one.”
Family knowledge and social norms: beliefs about food, physical activity, screen-based behaviors, 13 (33%)	WIC	24. “I think they think that anything besides watching TV is physical activity.”
	Early care and education	25. “I can count literally on one hand how many times a parent or a family member has come up to us, or me particularly, and said, ‘All right, I’m concerned about my child’s weight.’ So that doesn’t happen frequently at all, but it has happened.”
	Afterschool programs	26. “No, our parents really don’t seem concerned because no parents ever called or anything seeing about the physical activities that we do at our program. They know that we do them, but nobody ever comes with concerns, especially about their child.”
Family knowledge and social norms: knowledge of healthy lifestyles, 13 (33%)	WIC	27. “They [parents] are thinking that WIC juice is OK . . . . They are thinking that there is . . . good juice and bad juice. . . . They are thinking if it’s good juice they can have it all the time.”
Family knowledge and social norms: distrust of health care providers, 8 (21%)	WIC	28. “And we are like the bad guys . . . and they [parents] don’t want to hear anything about nutrition, anything about overweight. Because you are talking about something the doctor doesn’t even mention.”
	Primary health care	29. “The feedback I was getting was that: ‘School is for education, why the nurses concern about my child’s weight?’”
	WIC	30. “If the doctor doesn’t say anything, then they certainly don’t want to hear it from us.”
	Primary health care	31. “When she [a 12 year old girl[ came in, she took a scan of some of the staff and said: ‘How can you tell me to be healthy when your staff look like that?’”
Social disparities and chronic stress: food insecurity, 34 (87%)	Primary health care	32. “How was I gonna take that mom and the child . . . up to the nutritionist and tell her how healthy she should be eating when they don’t have any food?”
Social disparities and chronic stress: economic or employment stress, 34 (87%)	Afterschool programs	33. “When you’re on a fixed income and you only have so much money in food stamps you buy what you can. It’s probably not the most healthy choices.”
	Primary health care	34. “Then even small amounts of money are huge hurdles for these guys, you know? The entrance fee for the town soccer league is overwhelming.”
Social disparities and chronic stress: competing priorities, 22 (56%)	School	35. “They’re not thinking about what a nutritional meal's going to be. They don't think about nutritional meals. They're thinking about finding a meal.”
	Primary health care	36. “Families who are just trying to figure out a place to live and a way to make any kind of money to make ends meet are just not . . . they don’t have the energy to be concerned about this issue.”
Social disparities and chronic stress: lack of parental sense of control, 9 (23%)	Primary health care	37. “We see these new patients coming in and this is: ‘Oh, I’m coming in because . . . the pediatrician told me that my child was overweight.’ . . . and then the parents are bringing the child thinking the visit is just gonna be for the child.”

Abbreviation: WIC, Special Supplemental Nutrition Program for Women, Infants and Children.

a Values are the number and percentages of stakeholders who addressed the FEM construct.

b Providers are any stakeholders who provide health information.

### Family Ecology

**Family history and structure.** Within this subdomain, parent education and ethnicity–cultural background were mentioned by most stakeholders as affecting parents’ engagement or participation in childhood obesity prevention. Specifically, 32 (82.0%) out of 39 stakeholders, discussed parent education as a barrier, and 13 (33.3%) out of 39 stakeholders referenced ethnicity–cultural background as shaping cultural norms that could negatively affect parents’ engagement in obesity prevention.

The main cultural influence cited by 10 (26%) of 39 stakeholders representing all sectors was Hispanic families’ belief that high body weight is healthy. Participants reported that parents whose families recently immigrated to the United States were proud of their “chubby” children, and saw them as evidence of their ability to provide food (Table 2, quotes 1, 2); this concept is important because many families faced food insecurity in their home countries. Five stakeholders (13.0%) mentioned that grandparents’ beliefs that heavy babies are healthy also greatly influenced families’ daily routines (Table 2, quotes 3–5). Nine stakeholders (23%) from schools, afterschool programs, and health care discussed parents’ language and literacy needs, which are examples of parents’ ethnic–cultural background and education (Table 2, quotes 6–8). Although stakeholders reported addressing these needs by providing bilingual materials and a translator during appointments, these efforts were described as insufficient for fostering parent engagement (Table 2, quote 9).

**Organizational factors.** Fifteen stakeholders (39.0%) agreed that childhood obesity is a sensitive topic and that a good relationship between families and staff in key institutions is important when addressing obesity with families. Fourteen stakeholders (36.0%) representing almost all sectors, but especially from WIC, mentioned that health care providers lack the time to adequately address healthy behaviors because of parents’ need to discuss competing problems. Cultural competency and provider empathy were also described as playing a role. Additionally, stakeholders stressed that overall health should be addressed rather than overweight or obesity (Table 2, quotes 10–12).

**Community factors** *.* The most important community-level barrier reported by 19 (49.0%) stakeholders was the lack of safe neighborhoods. Safety concerns, including high traffic areas, unsafe sidewalks, and fear of violence, were all mentioned as barriers preventing children from going outside to play (Table 2, quotes 13, 14). Sixteen stakeholders (41.0%) across all sectors also described lack of transportation as a barrier. Although afterschool programs and sports clubs were offered free, stakeholders reported that families cannot attend as long as they lack consistent access to transportation (Table 2, quote 15, 16). Finally, 15 stakeholders (39.0%) across sectors mentioned that low-income families do not have access to affordable, healthful food (Table 2, quotes 17, 18).

**Media and policy factors.** Seven stakeholders identified media and policy factors, specifically marketing, as a barrier. For example, stakeholders mentioned that advertisements are confusing and irritating and perpetuate the misconception that healthful eating is expensive (Table 2, quotes 19, 20).

### Family Social and Emotional Context

**Family knowledge and social norms.** Thirteen stakeholders (33.0%) named different beliefs about food and physical activity as a barrier. For example, limited nutrition knowledge makes it more difficult for parents to make healthy food decisions (Table 2, quotes 21–23); another perceived barrier is parents’ belief that any activity except television viewing is physical activity (Table 2, quote 24). Stakeholders noted that asking parents more detailed questions about their children’s physical activity often revealed that parents did not actually know what physical activity meant. Consequently, stakeholders reported changing their counseling sessions to include examples of physical activity and different strategies for increasing children’s heart rate through physical activity. A total of 26 stakeholders (67.0%) mentioned that parents are often unaware or unconcerned about their child’s weight and do not engage in specific efforts to address obesity in their families. Stakeholders from WIC, health care, and early education reported that parents generally do not see weight gain as a problem (Table 2, quotes 25–26). Additionally, 1 WIC stakeholder said that parents believe that the products distributed through the WIC voucher program (eg, juice, milk, breakfast cereals, cheese, fruits and vegetables, peanut butter [20]) are healthful, and therefore, believe they can consume as much as they want (Table 2, quote 27).

Eight stakeholders (21.0%), mainly from WIC, reported parental distrust in stakeholders’ knowledge related to nutrition, physical activity, and body weight, especially if the child’s doctor did not address weight problems (Table 2, quote 28). Furthermore, 5 stakeholders (14.0%) felt that parents often saw advice related to their children’s weight as an intrusion into the way they raise their children (Table 2, quotes 29, 30). Several stakeholders also noted that parental distrust may stem from parents observing organizational staff consuming unhealthful foods and having weight problems themselves (Table 2, quote 31).

**Social disparities and chronic stress** *.* A total of 34 stakeholders (87%), identified families’ economic situation, that is, their inability to afford healthful foods and attendance fees, as a significant barrier to healthy lifestyles. Stakeholders reported that low-income families tended to use the support they get through the Supplemental Nutrition Assistance Program to buy cheaper, less healthful foods to be able to afford more food (Table 2, quotes 32–34). Linked to families’ economic situation are competing priorities that overrule child obesity and health-risk behaviors. More than half of all stakeholders (22 [56%]), mentioned that families experience competing priorities (eg, homelessness, addiction, food insecurity, being uninsured). Stakeholders said that low-income families would consume whatever food they could afford regardless of whether it was healthful (Table 2, quotes 35, 36). Nine stakeholders from health care and WIC said that many parents lacked of a sense of control over, and responsibility for, their child’s weight (Table 2, quote 37).

### Differences by sector

Although there was general cross-sector agreement on barriers parents encounter in obesity prevention, a few differences emerged. Of 16 stakeholders who identified lack of transportation as a barrier, 6 were from the afterschool sector (38%). Half of all stakeholders (4 of 8, 50%) who reported parental distrust in the providers nutritional knowledge, especially when addressing children’s weight problems, were from WIC. Eight of 13 stakeholders (62.0%) who identified cultural influences as a barrier to obesity prevention were from WIC and health care. Finally, 9 of 15 stakeholders (60%) who cited the quality of the relationship between families and staff in key institutions as influential to successfully engaging parents in obesity prevention efforts were from WIC and health care. 

## Discussion

Multisector interventions are recommended to prevent obesity in children. The success of such interventions requires not only that stakeholders are sensitive to the challenges experienced by low-income families in the context of obesity, but that there is congruence across sectors in stakeholders’ understanding of these challenges. Results from this study illustrate that stakeholders participating in MA-CORD have an intricate understanding of barriers to obesity prevention and control experienced by low-income parents. Stakeholder reports reflected awareness of the broader contextual factors affecting families in addition to social and emotional dynamics within families that could cause families to engage in obesity prevention.

Although stakeholder reports generally overlapped with FEM, numerous key areas not specifically highlighted in FEM were mentioned. Examples include parents’ language or literacy levels and the cultural competency and empathy of health care providers, which include the use of acceptable terminology when discussing a child’s weight. These themes are distinct from constructs referenced in FEM, such as parents’ ethnicity–cultural background, quality of relationship with staff, and parents’ distrust of health care providers, and they have implications for intervention approaches. For example, stakeholders acknowledged that having materials in Spanish and offering translator services was insufficient to improve provider–parent communication. The challenges extended beyond parent cultural background and the general provider–parent relationship and probably involved parents’ health literacy levels and provider and organizational levels of cultural competency, thus highlighting the need to emphasize these subdomains in future applications of FEM.

Another important finding that emerged is the role that extended family members such as grandparents play in food selection and a child’s weight, especially in Hispanic families (21,22). Although the role of extended family members is subsumed in ethnicity–cultural background in FEM, it may not be sufficiently emphasized. Previous research illustrates that grandparents have a strong caregiving role in Hispanic families (23). In our study, community stakeholders repeatedly mentioned the effect that grandparents had on their ability to communicate healthy lifestyle practices to parents. Stakeholders also mentioned that parents acquire their knowledge of nutrition from their own parents. These results underline the importance of explicitly including extended family members in childhood obesity prevention and control when working in communities with a large number of Hispanic families.

Results from this study have numerous implications for practice. First, results suggest that stakeholders, particularly stakeholders focusing on childhood obesity prevention, may be appropriately aware of the challenges experienced by low-income families in the context of obesity prevention; thus, attempts to increase stakeholders’ awareness through education may not be a good use of resources. Resources could be directed toward increasing parents’ health literacy levels, ensuring organizational cultural competency, and explicitly including extended family members in health promotion. Findings also iterate the importance of addressing contextual and family-level barriers when planning and conducting new interventions. Offering free programs at times parents are unable to participate with their children or when no transportation is available limits participation.

This study has numerous strengths. Community stakeholders play important roles in multisector obesity interventions, but to date they have not been emphasized in research in this area. To the best of our knowledge, this is the first study to examine key stakeholders’ perceptions of barriers to engaging low-income families in childhood obesity prevention across multiple community sectors. An important aspect of this study is the inclusion of stakeholders representing 5 community sectors. The use of a qualitative approach also provided the opportunity to delve more deeply into stakeholders’ views regarding facilitators and barriers to obesity prevention. Although interview questions were broad and open-ended and did not explicitly prompt theoretical constructs, results were highly consistent with the underlying theoretical model, lending further credibility to the results.

Despite these strengths, there are also numerous limitations, which need to be considered when interpreting the results. Because the study was nested within MA-CORD, stakeholders may have already had a strong interest in childhood obesity and may not represent stakeholders in other low-income communities. In addition, some intervention sectors were underrepresented; therefore, study findings may not fully represent the views of all stakeholders in these communities. Finally, stakeholder training had been initiated in some sectors at the time of the interviews. Although training sessions did not focus on barriers to obesity prevention experienced by low-income families, they may have increased stakeholder awareness of childhood obesity, thereby influencing interview responses.

This study adds to the literature by capturing the perceptions and experiences of key stakeholders across community sectors regarding barriers that low-income parents encounter when engaging in childhood obesity prevention. Findings illustrate stakeholders’ holistic awareness of the complexity of factors affecting families in the context of childhood obesity prevention and the consistency of those perspectives across community sectors. These results are encouraging because they suggest that some of the fundamental building blocks of multisector interventions for obesity prevention for vulnerable children and their families may already be in place. Resources may be more appropriately directed toward increasing parents’ health literacy levels, ensuring organizational cultural competency, and explicitly including extended family members as program targets in health promotion.

## Figures and Tables

**Table:** 

**1. The activity supported the learning objectives.**				
**Strongly Disagree**				**Strongly Agree**
1	2	3	4	5
**2. The material was organized clearly for learning to occur.**				
**Strongly Disagree**				**Strongly Agree**
1	2	3	4	5
**3. The content learned from this activity will impact my practice.**				
**Strongly Disagree**				**Strongly Agree**
1	2	3	4	5
**4. The activity was presented objectively and free of commercial bias.**				
**Strongly Disagree**				**Strongly Agree**
1	2	3	4	5

## Post-Test Information

To obtain credit, you should first read the journal article. After reading the article, you should be able to answer the following, related, multiple-choice questions. To complete the questions (with a minimum 75% passing score) and earn continuing medical education (CME) credit, please go to http://www.medscape.org/journal/pcd. Credit cannot be obtained for tests completed on paper, although you may use the worksheet below to keep a record of your answers. You must be a registered user on Medscape.org. If you are not registered on Medscape.org, please click on the "Register" link on the right hand side of the website to register. Only one answer is correct for each question. Once you successfully answer all post-test questions you will be able to view and/or print your certificate. For questions regarding the content of this activity, contact the accredited provider, CME@medscape.net. For technical assistance, contact CME@webmd.net. American Medical Association's Physician's Recognition Award (AMA PRA) credits are accepted in the US as evidence of participation in CME activities. For further information on this award, please refer to http://www.ama-assn.org/ama/pub/about-ama/awards/ama-physicians-recognition-award.page. The AMA has determined that physicians not licensed in the US who participate in this CME activity are eligible for ***AMA PRA Category 1 Credits*** ™. Through agreements that the AMA has made with agencies in some countries, AMA PRA credit may be acceptable as evidence of participation in CME activities. If you are not licensed in the US, please complete the questions online, print the AMA PRA CME credit certificate and present it to your national medical association for review.

## Post-Test Questions

### Article Title: Community Stakeholders’ Perceptions of Barriers to Childhood Obesity Prevention in Low-Income Families, Massachusetts 2012–2013

**CME Questions**

1. You are seeing an 11-year-old girl whose age-adjusted body mass index exceeds the 95th percentile. What was the *most* salient barrier to reducing obesity in the domain of family history and structure cited in the current study?
  - Siblings with obesity
  - Parents with obesity
  - Parent education
  - Cultural norms among racial and ethnic minorities
2. Which of the following stakeholder perspectives on cultural barriers to preventing childhood obesity was particularly relevant in the current study?
  - Hispanic families' belief that higher weight is healthy
  - Caucasians' overuse of commercially prepared foods
  - Preference for high-calorie foods among African Americans
  - Disregard for exercise among Native Americans
3. What was the *most* important community factor contributing to childhood obesity cited by stakeholders in the current study?
  - Lack of direct access to healthy foods
  - Inadequate green space for exercise
  - Lack of afterschool programs
  - Lack of safe neighborhoods
4. Which of the following statements regarding other variables related to childhood obesity in the current study is *most* accurate?
  - Parents generally had a good understanding of what defined physical activity
  - The majority of stakeholders believed that parents were aware of obesity issues and actively worked to address them
  - Stakeholders from the Special Supplemental Nutrition Program for Women, Infants, and Children were particularly inclined to cite parental distrust of nutrition advice
  - Child engagement and parental interest were particular problem areas reported by stakeholders in afterschool programs

**Evaluation**
